# Intelligently Taking Out Universal Screws and Nail Caps After Spine Internal Fixation

**DOI:** 10.1111/os.12488

**Published:** 2019-08-20

**Authors:** Ye Zhang, Ming‐Ju Sun

**Affiliations:** ^1^ Department of Orthopaedics 313rd Hospital of People's Liberation Army Huludao Liaoning China

**Keywords:** Novel technique, Pedicle screw, Spinal fixation

## Abstract

The purpose of this study was to present a surgical technique for taking out universal screw and nail caps which were difficult to removed. We used a variety of industrial hex wrenches, dental drills, and other equipment to take out internal hex nuts with different specifications (32 pieces) and universal screws (15 pieces) in 28 patients. A total of 32 nuts were taken out, 3 of which were polished by the industrial drill. A total of 17 were spun by hand, 2 were spun by locking pliers, 10 were turned by “I” type screwdriver, and 3 were turned by bone blade. A total of 15 screws were taken out, 9 of which were removed with a wrench and the other 6 by means of locking pliers after re‐fixing with a truncated titanium rod. The novel technique is simple and provides a solution following failure of a supporting device.

## Introduction

The pedicle screw internal fixation system is widely used for the treatment of spinal fractures and degenerative instability^1^, ^2^, ^3^. However, there are many potential complications, such as pedicle screw breakage, loosening, and deformation^4^. Pedicle screw loosening or breakage is a common complication, and depends on the strength of the interface between the internal fixation device and the bone^5^, ^6^. The extension of the internal fixation device also increases the risk of breakage from screw fatigue^7^. Therefore, most screws need to be removed. In the past, the removal of such screws required finding the manufacturer of the original equipment to source appropriate tools or using imitation tools. Due to the large number of manufacturers, the designs of pedicle screw tails and the nut specifications vary^8^. It is often difficult to find suitable equipment to remove them, and some patients cannot provide details of manufacturers of the screws. If the grooves are twisted, slippery, or deformed, this will increase the difficulty of taking out the screw. There are reports on the removal methods of broken screws, and each method has its own advantages and disadvantages^9^, ^10^, ^11^, but there are no reports in the literature on removal methods in circumstances such as the screw cap slipping, deformation, and damage. We use a variety of industrial hex wrenches, dental drills, and other equipment to take out imported and domestic internal hex nuts (32 pieces) and universal screws (15 pieces) with various specifications. Effective solutions to the above problems are described below.

## Materials and Methods

### *Patients' Information*

The 28 patients in this study were aged between 33 and 62 years, with an average age of 43 years. Among them, there were 22 men and 6 women, 16 with spinal fractures and 12 with degenerative diseases of the spine. The operation time ranged from 10 to 83 months after the last operation. Internal fixation sites: T_10_–T_12_ in 3 cases, T_11_–L_1_ in 4 cases, T_12_–L_2_ in 8 cases, L_3_–L_5_ in 5 cases, and L_4_–L_5_ in 8 cases. Fractures had healed and bone graft fusions were intact in all patients.

### *Surgical Techniques*

All patients were operated on in prone position under general anesthesia, and internal fixation screws were exposed through conventional posterior midline incision. This study focused only on the screw removal procedure.

#### *Removal of the Nuts*

**Figure 1:**
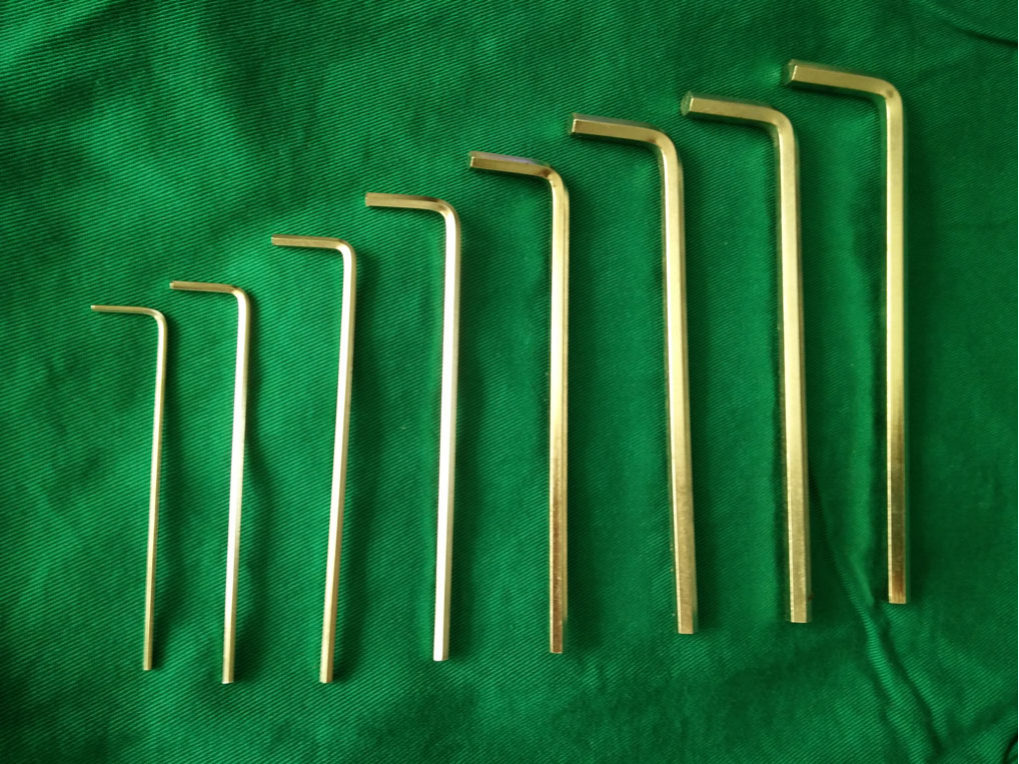
Industrial‐sized hex wrench.

**Figure 2:**
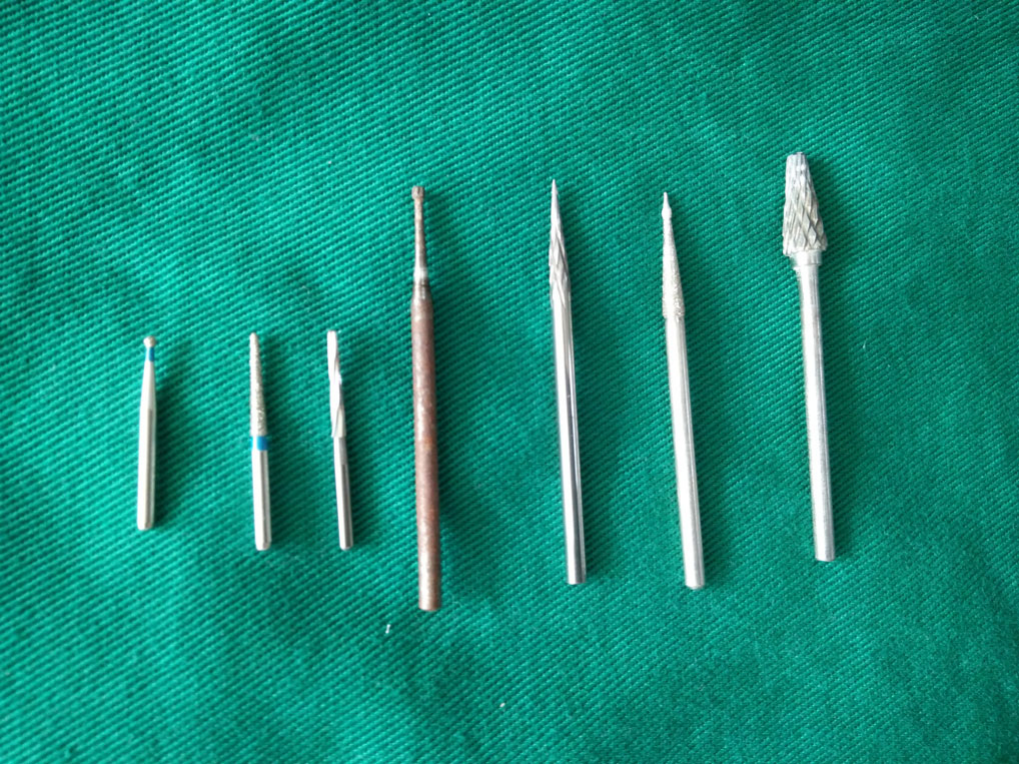
Dental drill.

**Figure 3:**
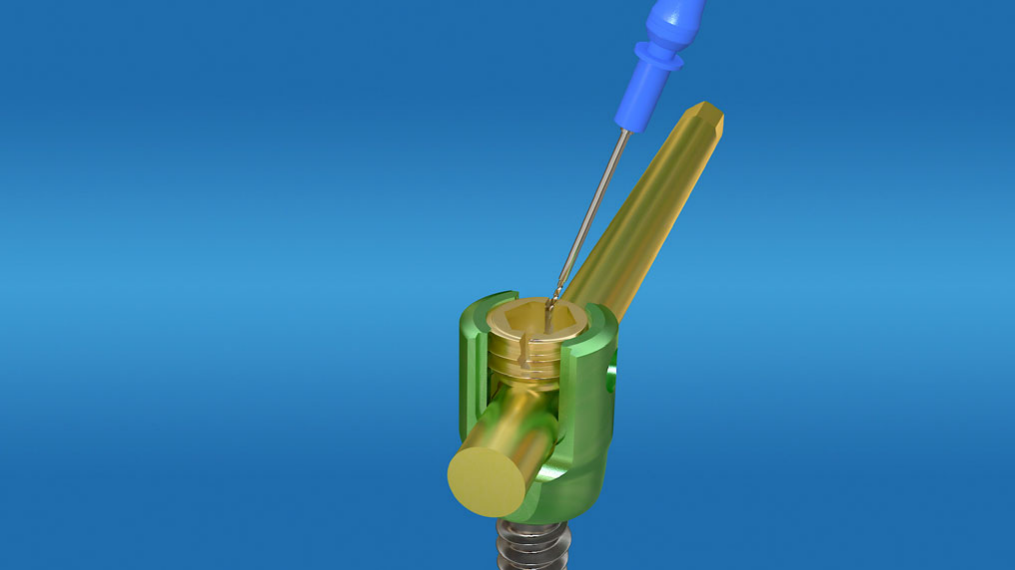
Grinding cap of the nail.

**Figure 4:**
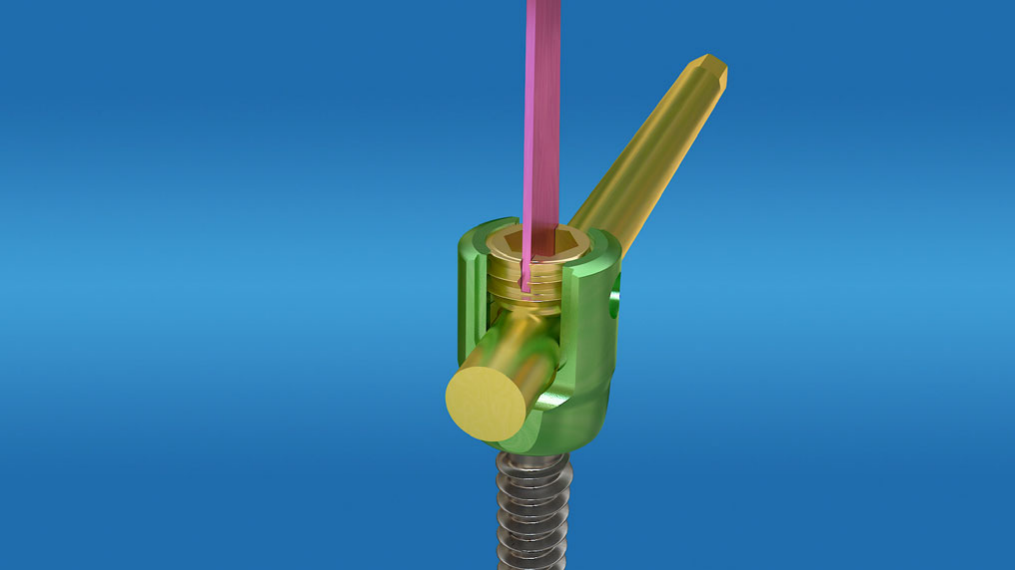
Turn the cap of the nail.

A wide variety of industrial‐sized hex wrenches (Fig. 1) were sterilized and ready for use. After the pedicle screw was exposed, the scab or scar tissue in and around the groove of the nut was cleaned, and the best matched groove wrench was selected and inserted. The nut was loosened once by hand or the other arm of the inner hexagon wrench was clamped in parallel with the forceps to increase the rotational force of the arm to rotate the nut. If the hexagon groove of the nut was slippery and deformed, different types of (disinfected) dental drills (Fig. 2) were used. The dental drill was assembled on the electric drill, before grinding both sides of the nut between the two arms of the nail tail (Fig. 3) to reach the bottom of the nut groove, and forming the “I” type notch, and then loosening the nut with a suitable “I” type screwdriver or a bone blade (Fig. 4). A total of 32 nuts were taken out, 3 of which were polished using the industrial drill; 17 were spun by hand, 2 were spun using locking pliers, 10 were spun using an “I” type screwdriver, and 3 were spun by bone blade.

#### *Removal of the Universal Screws*

**Figure 5:**
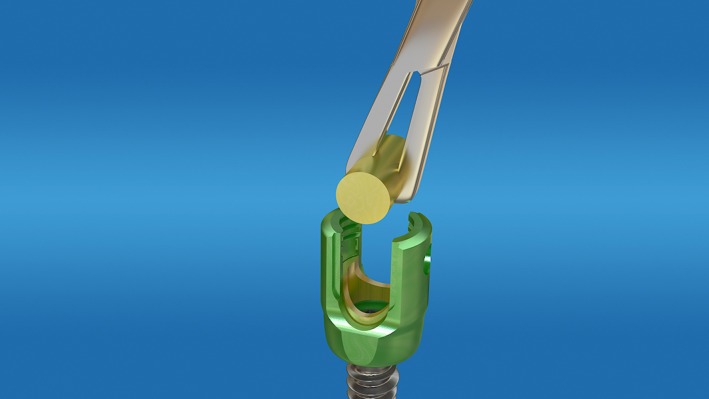
Insert the rod into the nail tail.

**Figure 6:**
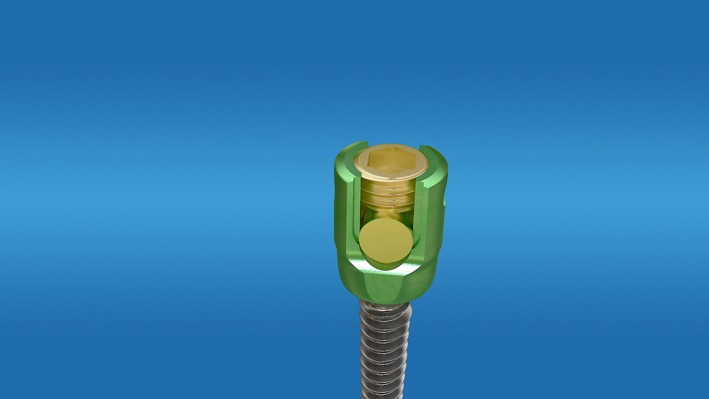
Consolidate the nail tail and the nail body.

**Figure 7:**
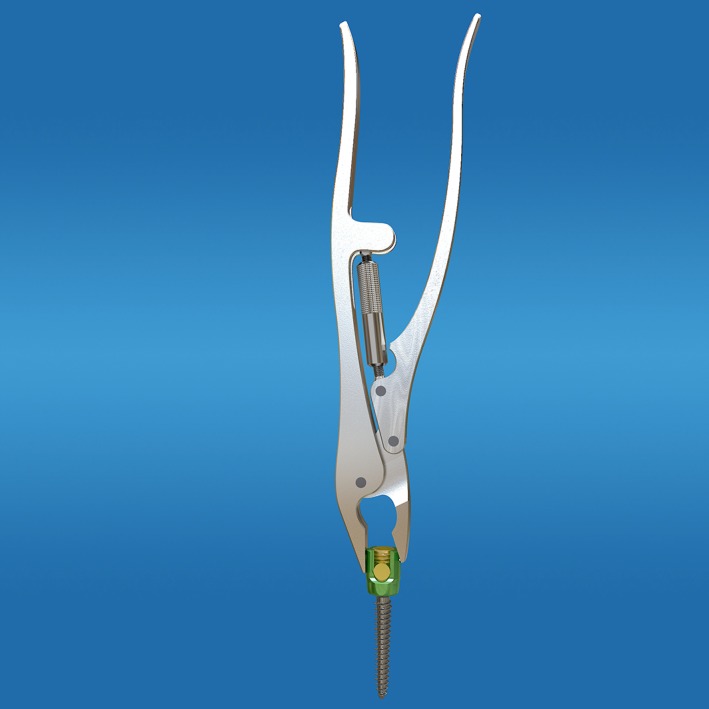
Remove the screw by locking pliers.

A wrench was selected that best matched the hexagon in the groove. It was placed in the groove at the end of the screw to loosen the screw. If the hexagonal groove in the tail of the screw had slid or was covered by bone tissue, we cut the titanium rod which had been removed earlier to approximately 1 cm with a rod breaker device, which is a commonly used instrument for spinal internal fixation surgery, and selected a nut to fix the 1‐cm titanium rod firmly in the groove of the end of the screw (Fig. 5), consolidated the nail tail and the nail body into a whole (Fig. 6), clamped the two arms of the screw by locking pliers (Fig. 7), and then removed the screw. A total of 15 screws were taken out, 9 of which were removed with a wrench, and the other 6 pieces were removed by means of powerful forceps after re‐fixing with a truncated titanium rod.

## Results

All screws were completely removed. All patients in this study were followed up, with a follow‐up time between 11 and 24 months, and an average follow‐up time of 13 months. All the postoperative incisions healed well. All patients were followed up with X‐ray and CT examination after surgery, indicating good bone healing at the nail tract, no foreign body residue, no pain in the spine with good activity, and no complications of infection and nerve injury.

## Discussion

The method is easy to implement and is a novel technique that has not been described previously in the published literature. It provides a solution following failure of a supporting device. However, grinding the nail will prolong the operation time. If a new type rod of breaker instrument that is similar to titanium mesh scissors can be designed, the titanium rod could be cut off directly by the tail of the screw without increasing soft tissue damage.

Attention should be paid to the application process: (i) the inner hexagon wrench should be placed at the lowest position of the nut groove and it is best matched with the inner hexagon, and rotating the wrench should not be done with force to avoid causing the screw groove to slip; (ii) when grinding the nut with the dental drill from the inside out, use its side to grind the groove wall of the nut or tilt the drill from the top down, and choose between the two arms of the nail tail; (iii) the industrial hexagon wrench and dental drill should be updated timely to avoid screwing pedical screw nut slippery and breaking dental drill when grinding; and (iv) when grinding the two sides of the nut, the upper and lower openings of each side should be as wide as possible, and the screwdriver or bone blade should be changed if necessary during the grinding process to avoid openings that are too wide on both sides of the nut.
